# Utilization Rates and Perceptions of (VCT) Services in Kisii Central District, Kenya

**DOI:** 10.5539/gjhs.v5n1p35

**Published:** 2012-11-01

**Authors:** Epule Terence Epule, Moto Wase Mirielle, Changhui Peng, Balgah Sounders Nguh, Josephat M. Nyagero, Alice Lakati, Ndiva Mongoh Mafany

**Affiliations:** 1 University of Quebec in Montreal, Institute of Environmental Sciences, Montreal, Quebec, Canada; 2 Faculty of Medicine, Department of Public Health, Lund University, Lund, Sweden; 3 University of Buea, Department of Geography, Cameroon; 4 African Medical and Research Foundation, Health Programmes Development Directorate, Nairobi, Kenya; 5 Sitting Bull College, 9299 Hwy 24, Fort Yates, USA

**Keywords:** utilization, voluntary counseling and testing, married persons, HIV/AIDS

## Abstract

Voluntary counseling and testing (VCT) services have been set up in most Districts in Kenya due to the rising surge of HIV/AIDS. However, the use of these services among married persons has not been fully explored. In Kissi, the issue of VCT is pressing as the rate of HIV prevalence is close to 3%. In 2006, about 20 000 clients came for VCT services in Kenya yet only 165 of these were married persons. In the Keumbu sub-district hospital, of the more than 1000 clients that came for VCT services, approximately 29% were married persons. This paper therefore aims at determining the utilization of VCT services by married persons in the study area. The qualitative data was obtained principally through two focus group discussions (FGDs) in which the respondents were asked to comment on their use of VCT services while the quantitative data was obtained from interviews with 245 respondents. The qualitative data was analyzed through verbatim transcription while for the quantitative data; the responses were coded and populated into SPSS from which the frequencies and percentages were calculated. The results show that actual use of the VCT services is low (28.1%) but slightly higher among female respondents than males. The low usage may be attributed to (a) fear of results, (b) death anxiety, (c) lack of confidentiality and lastly, (d) fear of stigmatization. Female respondents were found to have a greater awareness of VCT and thus its potential use.

## 1. Introduction

Voluntary counseling and testing (VCT) can be described as a situation in which people go in for counseling which informs them properly on how to handle all possible outcomes of testing for HIV (Hackney, 2002; Wolff et al., 2005). VCT is a widely accepted approach used in the prevention and control of HIV/AIDS (Wolff et al., 2005). Knowledge about one’s HIV status is an important aspect as far as the prevention of HIV infection is concerned because those who test negative will guard against infection while those who test positive will seek treatment and care services (Nuwaha et al., 2002; Adebajo et al., 2002). However, knowledge of HIV status is still low as the majority of those who are aware of the existence of HIV/AIDS have not been tested (Nuwaha et al., 2002; Adebajo et al., 2002). It is therefore worth noting that knowledge of one’s HIV status alone remains inadequate since those tested still need to be educated on the potential benefits of early diagnosis and treatment (Hackney, 2002; Kalichman, 2003).

It has been found that lack of proper knowledge and awareness on the benefits of VCT greatly reduced the number of individuals who would have been willing to undertake VCT (Nuwaha et al., 2002). It is important to note that going for counseling does not often lead to testing on the same day since the person involved has to be properly informed on how to handle the possible results; the reason for the discrepancy between counseling and testing in most statistics (NASCOP, 2001; Nuwaha et al., 2002; Yates et al., 2002). The Kenyan demographic and health survey is also argued to have showed a universal knowledge of HIV/AIDS with almost no difference by age, marital status, urban-rural residence or level of education, yet, the number of people who go for testing is little (Maman et al., 2007; Sheri et al., 2006; Hesketh et al., 2005).

The burden of HIV/AIDS continues to pose a major challenge to Kenya’s health care system. It is estimated that about 1.5 million Kenyans are HIV positive with about 900,000 between the ages of 15-49 and 66,000 younger than 14 years. About 1.2 million children have been orphaned by AIDS and in 2009 about 80,000 people died from AIDS related diseases in Kenya (NACC, 2007; Hesketh et al., 2005; UNAIDS, 2010). The demographic and health survey showed that only about 14% of adults had tested for HIV when compared to the Kenyan population of 40 million (CBS, 2003; Sherr et al., 2007). This indicates that only a small fraction of Kenyans at risk have undergone VCT services, yet, a majority are aware of these services. The Kenyan government, in an attempt to improve on the uptake of VCT services had established to date more than 600 functional VCT service centres by June 2006. These included those at Government of Kenya (GOK) health facilities, faith based organisation facilities, community based facilities, stand alone centres as well as the introduction of mobile testing services (NASCOP, 2007; Pool et al., 2001; Lorrainea et al., 2007).

The HIV prevalence in Kisii is estimated at about 3.0% (NACC, 2007). The total number of VCT clients in the district in 2006 was about 20,483 with 16.5% of these being married persons (NASCOP, 2007). The Keumbu sub-district hospital VCT statistics show that of the 1446 clients who come for testing since its establishment in 2006, 432 (29.8%) of them were married. Married persons often tend to believe that they are not at risk of HIV, and therefore they see no need to go for testing. It has been shown that among married persons who came for VCT, 80% were concordant while 19.7% were discordant (Alwano et al., 2003; Yates et al., 2002). Among married persons with at least one HIV infected partner, 40.8% had a discordant HIV uninfected partner. As such, the rationale of this paper is to bridge the knowledge gaps by investigating what respondents think about VCT. The objective is to attempt an analysis of the perceptions and rates of utilisation of VCT services among married persons in the study area. The analysis of the use of VCT services among married persons is vital because in 2008 about half of all new HIV/AIDS infections were transmitted during heterosexual sex in married relationships while 20% was during casual heterosexual sex (UNGASS, 2010).

## 2. Methods

### 2.1 Study Design and Population

A Cross–sectional design was used to determine the utilization of VCT services in Kiogoro division. The study combined both qualitative and quantitative methods in collecting data to complement each other in other to provide detailed information to attain the objectives (Yates et al., 2002).

The study targeted married persons/respondents who were residents of Kiogoro division as people in marital unions often feel they are free from HIV infection and so do not go for VCT services (Alwano et al., 2003; Yates et al., 2002). Only married persons whose partners were alive were included in the study since one of the variables included knowledge of partners using the VCT services as well as taking advice from partner to go for the services. All widows, widowers and separated couples were thus excluded from the study (Bryman, 2004).

### 2.2 Study Area Selection and Sampling Procedure

The broader study area in which this study was carried out is called Kisii Central district. It was selected because of its high HIV prevalence, estimated at about 6.01% while the national rate as of 2006 was 6.9% (NASCOP, 2007; NASCOP & Ministry of Health, 2006). Kisii has several administrative units which are: Keumbu, Suneka, Mosocho, Masaba, Masimba, Kiamokama, Marani, Ogembo, Irianyi, Kisii municipality, Nyamache, Bongo and Kiogoro. In all these units, Kiogoro, with an area of 61.3 km^2^ and a population of about 32,638 people, was selected. Kiogoro is served by the Keumbu sub-district hospital which has a VCT centre, good road network and proximity to Kisii municipality. Kiogoro has two locations which are Kegati and Kiogoro locations. This study was carried out in Kegati with a population of about 15,000 people, about 7,000 males and 8,000 females (NACC, 2007).

Selection of households in Kegati was done randomly by the toss of a coin and all its three sub locations were included in this study. Fourteen villages out of forty five were sampled randomly using the ballot method. This involved assigning codes (by numbering) to all forty five villages (Bryman, 2004). The numbers were written on small pieces of paper, folded, shuffled and then fourteen picked at random in succession without replacement and with shuffling done after each draw. The chosen numbers were then decoded to reveal the names of the villages. Once an inaccessible village was drawn, it was replaced by another randomly selected village till the desired number was reached.

### 2.3 Determination of Sample Size

The study utilized the statistical formula for population surveys to determine the sample size.

This is given by: n= Z^2^pq/d^2^

Where n is the desired sample size (population >10,000), Z is the standard normal deviate set at 1.96 corresponding to a 95% confidence interval, P is the proportion of the target population estimated to have used the VCT services. If we go by the statistics that a proportion of 14.8% men and women had gone for VCT services in Nyanza Province (CBS, 2003), then, P is 0.148.

q= 1-p i.e.1-0.148=0.852

d is the degree of accuracy desired, set at 0.05

The desired sample size for the study is given by:

n= (1.96) ^2^ (0.148) (0.852)/(0.05)^2^

Minimum desired sample size is 194, actual n is 245 (number of respondents interviewed).

### 2.4 Data Collection Procedure and Tools

Through face to face interviews at household level, quantitative data was obtained from a total of 245 respondents. These interviews only started after getting their verbal consent. Qualitative data was obtained through focus group discussions (FGDs) and in depth interviews. Two FGDs were conducted, each group had 12 participants, 6 males and 6 females and they were free to express themselves on the topic under discussion. The participants for the FGDs were invited using the Snowball approach. The area chief identified one suitable couple in each of the sub-locations; this person identified the next person until the required number of respondents was obtained. Three in-depth interviews were conducted with the head of the Keumbu VCT center, the counselor at the VCT center, the local administrator (chief) of Kegati location, using in-depth interview guides. They were informed in advance on the time and place of interview (Smith, 2003).

The topics included in the FGDs were geared towards exploring if the respondents had ever gone for VCT? Where they obtained the VCT services? Did they go for HIV testing after the VCT? What reasons were behind their going for VCT and/or not going for VCT? What are the benefits of VCT? The FGDs and in-depth interviews were conducted using guides with all the topics above well stated. Tape recording was completed during the FGDs to compliment the information collected by note taking. The FGDs were moderated by the principal investigator who had research assistants that helped in cases of language difficulties.

### 2.5 Quality Control

A clear inclusion and exclusion sampling criteria was consistently used for the selection of the respondents. Research assistants were also carefully selected based on educational background and sound knowledge of the area selected for the study. Assistants were necessary since the principal investigator was not familiar with the local language (*Ekegusii*). Each research assistant was given a field work manual which included the purpose of the study, sampling procedures and instructions for administering the interview schedules. Brief intensive training consisting of interview techniques with emphasis on; approaching a respondent, administering of interview schedules and ethical issues. All unclear and ambiguous questions as well as skip patterns were explained during the training session. The purpose of this was to minimize errors during data collection (Bryman, 2004).

Interview schedules were pre-tested after training using members of the community in the neighboring Keumbu division who exhibited similar characteristics as the targeted population. The assistants were closely supervised during the data collection process. At the end of each day, all interview schedules were checked for completeness of questions and consistency in answering of questions. Field spot checks were also completed to ensure that the sampling procedures were adhered to. Tools were thoroughly numbered and stored appropriately by the researcher. The FGDs were moderated by the principal investigator with the help of research assistants who translated the information to ensure the maintenance of quality. Frequencies were run during data cleaning to check for double entries, missing values and inconsistencies in order to minimize data entry errors.

### 2.6 Ethical Considerations

At the district level, permission was obtained from the Medical officer of Health, the District Commissioner and the District Education Officer. At the divisional level, permission to proceed with the study was sought from the Divisional Officer, the Chief and his assistants who informed the village heads about the study. Furthermore, this study was also authorized by the Ministry of Science and Technology of Kenya. The participant’s verbal consent was sought before commencement of the interview and the married persons/respondents were assured of the right to deny consent without any reprimand (Sherr et al., 2007). The respondents were always reassured each time the study questions moved to very sensitive issues and of the confidentiality of their responses. The research team remained respectful of the rights of the respondents, ensuring their culture was respected.

### 2.7 Data Analysis

The collected data was coded and entered into an excel sheet and later populated into the Statistical Package for Social Sciences (SPSS) version 19. Data cleaning was done to ensure that there were no missing values or double entries by running of frequencies. Further checks were done to ensure that the missing cases were valid. The collected numerical data was analyzed using frequencies and percentages. Analysis of the FGDs and in-depth interviews involved verbatim transcription of tapes into well organized set of information using a thematic matrix. Verbatim transcription involved the direct translation of the responses of the respondents into English from the recorded tapes and notes. The thematic matrix approach involved transcription based on the primary theme, secondary theme and tertiary theme (Yates et al., 2002).

## 3. Results and Discussions

### 3.1 Ever Gone for VCT?

**Figure 1:**
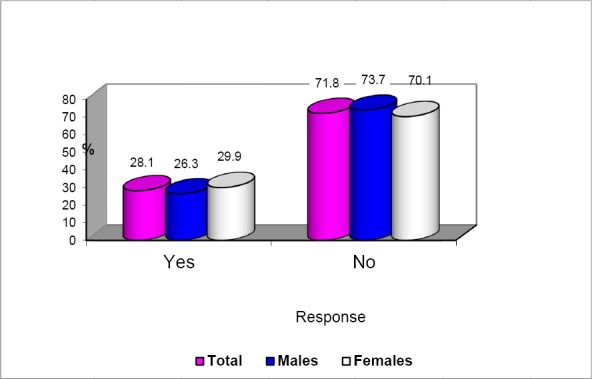
Percent distribution of utilization of VCT services by gender (n=245)

Even with high levels of awareness of VCT services (99.6%), very few respondents had actually made active use of VCT services. Only 28.1% of respondents had gone with slightly more females (29.9%) than males (26.3%). Almost three quarters (71.8%) of respondents interviewed had not gone for the services with slightly more males than females (73.7% males, and 70.1% females) (Figure 1). The Kenyan demographic and health survey of 2003 reported utilization of 14.75% and 15.2% for Nyanza province and National level respectively (CBS, 2004). These findings suggest a significant increase in utilization of the VCT services probably due to increased sensitization and awareness of the services. The FGDs revealed that, with the constant increase of HIV among married persons, married persons sought ways of changing behaviour, one of which is counseling and testing services in hospitals. One participant shared his view: *“These past years, most couples who have lost a partner in this community have been due to HIV-related illnesses. Even though they believed not to be at risk, they have now resorted to Counseling and testing services in order to avoid infecting each other unknowingly”*.

### 3.2 Place Obtained VCT Service

About 95.7% of the 69 respondents that had obtained VCT services got the services from public health facilities while about 4.3% obtained VCT services from the private clinics. The majority of respondents (65.2%) obtained the VCT services from Kisii district hospital located 5 kilometres away from the study area. Keumbu sub-district hospital which is very close to the study area had served about 13.0% of the married persons, probably because it is in the same community and poses problems of confidentiality. As such, some respondents went as far as Nairobi to receive these services (Table 1).

**Table 1:** Distribution of service delivery facilities where VCT services were obtained (n=69)

Public/GOK facilities	Frequency	Percent
Kisii	45	65.2
Keumbu	9	13.0
Kenyatta hospital	2	2.9
Kiogoro	2	2.9
Oresi	2	2.9
Keroka	1	1.4
Others	5	7.2
**sub-total 1**	66	95.7
**Private**		
Bosongo	2	2.9
Ram	1	1.4
**sub-total 2**	3	4.3
**Total**	69	100

The finding above strongly suggests an issue of confidentiality as it was confirmed by a female respondent during an FGD that *“we don’t go to Keumbu hospital because we do not want to be said to be having HIV by members of our community who visit there all the time”*. It was further stated by various respondents that they trusted/preferred the services at Kisii district hospital more than those at Keumbu. The counsellor at the VCT centre revealed that *“there is usually a massive turnout during mobile VCTs than at the hospital because those who do the testing at these sites do not know the community members”*. This further supports the argument that the people feel insecure in the “presence” of the patients seeking other health services who happen to know them. These findings are consistent with findings in various cited studies (Day et al., 2003; Wolff et al., 2005; Ugboga & Ademola, 2004; Sweat et al., 2000; Machekano et al., 2000) where respondents imagined their names being written and pasted on the walls of the counseling room for everyone to read.

### 3.3 Those Who Took the HIV Tests

**Figure 2:**
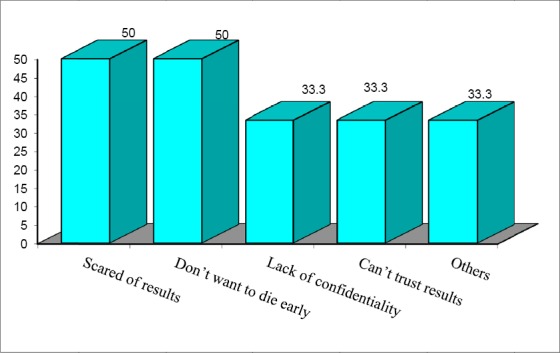
Percent distribution of reasons for not testing for HIV (n=69)

Among the 69 respondents (28.1%) who had gone for VCT services, 62 (89.9%) had later taken the HIV test while 7 respondents (10.2%) did not take the test for HIV. This notion of people going for VCT services and later not taking the test is fairly similar to those obtained during the Kenyan Health Survey (CBS, 2003; Sweat et al., 2000) which found that 85% of the respondents in Nyanza province and 83.7% at the National level visited the VCT centres but did not take the test for HIV. This indicates that not all respondents who go for VCT services end up testing for HIV. The majority of the respondents seem to change their mind after the pre-counseling session. In terms of gender, more females (94.7%) than males (83.9%) had tested for HIV in the study area. This is in conformity with previous studies such as (CBS, 2003; Rony et al., 2003; Ugboga & Ademola, 2004) which show that more woman than men took the test. Being scared of the results and feeling that knowing of their HIV positive status would make them to “*die early due to worries if found to be positive*” were the main reasons given by those who went to the VCT centre but declined to take the test (Figure 2).

Almost all of the 62 respondents who took the test got their results (96.8%), with two respondents not being courageous enough to face the results. The main reason why they did not want to know their results was fear of coping if found to be HIV positive. Similar results were reported in studies by Corbett et al. (2006) and Sweat et al. (2000) in which respondents felt they were incapable of coping with adverse life events if found positive as well as if testing was not linked to HIV care.

### 3.4 Reasons for Going for VCT

Almost three quarters of the 69 respondents (72.5%) who went for VCT services did so in order to know their HIV status while 21.7% obtained the services as a pre-marriage requirement. In terms of gender, more females (86.8%) mentioned that they went for the services in order to know their status compared to the males (54.8%). On the other hand, more males (32.3%) than females (13.2%) mentioned pre-marital requirements as a reason for testing. Among the other reasons mentioned, there was no great variation by gender suggesting that the respondents almost equally had similar reasons for going for the services. A small proportion (13.0%) went for the services as a result of demand from the partner while other reasons mentioned included; (a) suggestion from relatives, (b) just to be counselled about HIV or (c) because they felt that their health was not good. A few respondents mentioned that they went for VCT services because it was a requirement from their employer, others wanted to know if their spouse had been faithful and some had to donate blood (Table 2).

**Table 2:** Percent distribution of reasons for going for VCT services (n=69)

Reasons	Males (n=31)	Females (n=38)	Total /%
To know status	54.8	86.8	50 (72.5)
Pre- marriage requirement	32.3	13.2	15 (21.7)
Demand from partner	12.9	13 2	9 (13.0)
Suggestion from relative	16.1	5.3	7 (10.1)
Poor health	12.9	7.9	7 (10.1)
To be counseled	6.5	10.5	6 (8.7)
Demand from employer	6.5	2.6	3 (4.3)
Others	16.1	13.2	9 (13.0)

*% exceeds 100 (multiple responses)*

During the FGDs there was a consensus about pre-marriage requirement of VCT services. The female respondents argued that, *“these days many brides and grooms must insist on going for VCT services before getting married so as to get counseling on healthy behaviours and other benefits of knowing one’s status”*. These reasons suggest that, the respondents are aware of the benefits of early testing and will therefore contribute towards behaviour change and eventually reduction in HIV prevalence. Various recent studies that support this view all argue that clients seeking VCT services are mostly those planning for marriage and not those who are already in marital union, further suggesting the need for married persons to be sensitized about their risk of HIV infection as well (Rony et al., 2003; Painter, 2001; Day et al., 2003; Adeneye et al., 2006; Nuwaha et al., 2002).

### 4.5 Reasons for Not Going for VCT

From the 176 respondents who had not gone for VCT, 48.3% felt that they trusted their spouses and had never been unfaithful to each other, so it was not seen as necessary going for VCT. Less than a quarter (20.5%) responded they were too busy while 13.1% mentioned that the VCT centre was very far, so they had not been able to go for testing. Fear of handling positive results was mentioned by 12.6% of the respondents. However, 2.8% argued that they had no idea of where to access these services. In terms of gender, there was not much variation among those who had not gone for VCT services and likewise their reasons for not going for VCT (Table 3). This is also consistent to the results of Wolff et al. (2005) and Day et al. (2003).

**Table 3:** Reported reasons for not going for VCT services (n=176)

Reasons	Males (n=87)	Females (n=89)	Total
Trust my spouse	48.3	48.3	85 (48.3)
No need	41.4	38.2	70 (39.8)
Very busy	19.5	21.3	36 (20.5)
Long distance	11.5	14.6	23 (13.1)
Fear handling positive results	9.2	15.7	22 (12.5)
Fear of stigma	8.0	13.5	19 (10.8)
Don’t want to know	6.9	12.4	17 (9.7)
Don’t know the importance	3.4	3.4	6 (3.4)
Don’t know where to get VCT services	2.3	3.4	5 (2.8)
Others	20.7	14.6	31 (17.6)

*% exceeds 100 (Multiple responses)*

The FGD revealed that female respondents in the community were not free to make decisions or suggestions concerning VCT services because most men seemed uninterested. This is relevant as the study area in question is a community in which men make all the decisions. One respondent said: *“I cannot go without him giving permission because if I do, he will ask; where are you going, are you not sure of me? This is especially since he is not interested”*. The female respondents also came to consensus that villagers only go to hospitals when they felt unwell. This therefore means that they don’t see a reason to go for VCT services when they don’t feel sick.

Female respondents also argued that most seminars where issues about HIV are discussed excluded them indirectly since their husbands would attend and leave them at home to take care of kids. As such, they don’t get the right information from their husbands when they return. An older female respondent sadly exclaimed; “*most seminars and barzas where these issues are discussed do not involve us so we tend to miss much. I think we should be actively involved”*. Other reasons mentioned were fear of discrimination and stigma if results had to be positive, since they indicated that most married persons were unfaithful to each other. The above mentioned reasons for not going for VCT services strongly compares with recent studies by Kalichman and Simbayi (2003), Sheri et al. (2006), Wolff et al. (2005), Rony et al. (2003) and CDC (2007) on fear related misconceptions about how VCT centres function, the test results, as well as fears of disclosure of extramarital relationships in case results were positive.

### 4.6 Future Use of VCT Services

From the 176 respondents who had never visited the VCT centres, 68.2% said they planned to go for VCT in future while 31.8% refused to make any future visit to the VCT centres. In terms of gender, slightly more females (73%) than males agreed to use VCT services in future. The desire to go for VCT in future was strongly supported in the FGD by women. According to Sheri et al. (2006), 71% of respondents confirmed their intentions to go within 6 months with reasons being; the availability and provision of treatment and if they were to test together as a couple. Rony et al. (2003) also discuss similar findings.

## 5. Conclusions

Utilization of VCT services tested low among married persons/respondents partly because they feel they are not at risk of HIV. Younger married persons utilized the services more than older married persons, resulting from their perceived higher risk of infection while the older married person believed the issue of HIV and screening is a new era that does not concern them. Female respondents were seen as having higher utilization of VCT.

Respondents who had never made use of VCT generally had positive attitudes towards future use of the services. Infidelity and perceived reasons for not going for VCT services coupled with the high HIV prevalence in the district suggests a significant number of married persons are at risk of HIV infection. The issue of respondents not knowing their spouses’ HIV status suggests a gap in communication which needs to be addressed in future research. More females also expressed the desire to visit VCT service centres in the future.

Married persons were found to go for VCT services at Kisii district hospital which is far from the study area instead of Keumbu, probably because they are less likely to be recognized by other patients seeking services. This suggests an issue of confidentiality, HIV related stigma and other factors that affect utilization.
